# Study of Prepared Lead-Free Polymer Nanocomposites for X- and Gamma-ray Shielding in Healthcare Applications

**DOI:** 10.3390/polym15092142

**Published:** 2023-04-29

**Authors:** Abdulrhman Hasan Alsaab, Sadek Zeghib

**Affiliations:** Physics Department, Faculty of Science, King Abdulaziz University, Jeddah 21589, Saudi Arabia; aalsaab0031@stu.kau.edu.sa

**Keywords:** radiation shielding, Bi_2_O_3_, polymeric nanocomposite, attenuation of gamma rays

## Abstract

Polymer composites were synthesized via melt mixing for radiation shielding in the healthcare sector. A polymethyl-methacrylate (PMMA) matrix was filled with Bi_2_O_3_ nanoparticles at 10%, 20%, 30%, and 40% weight percentages. The characterization of nanocomposites included their morphological, structural, and thermal properties, achieved using SEM, XRD, and TGA, respectively. The shielding properties for all synthesized samples including pristine PMMA were measured with gamma spectrometry using a NaI (Tl) scintillator detector spanning a wide range of energies and using different radioisotopes, namely Am-241 (59.6 keV), Co-57 (122.2 keV), Ra-226 (242.0), Ba-133 (80.99 and 356.02 keV), Cs-137 (661.6 keV), and Co-60 (1173.2 and 1332.5 keV). A substantial increase in the mass attenuation coefficients was obtained at low and medium energies as the filler weight percentage increased, with minor variations at higher gamma energies (1173 and 1332 keV). The mass attenuation coefficient decreased with increasing energy except under 122 keV gamma rays due to the K-absorption edge of bismuth (90.5 keV). At 40% loading of Bi_2_O_3,_ the mass attenuation coefficient for the cesium ^137^Cs gamma line at 662 keV reached the corresponding value for the toxic heavy element lead. The synthesized PMMA-Bi_2_O_3_ nanocomposites proved to be highly effective, lead-free, safe, and lightweight shielding materials for X- and gamma rays within a wide energy range (<59 keV to 1332 keV), making them of interest for healthcare applications.

## 1. Introduction

Radiation protection is a significant concern for patients, healthcare personnel, radiation field workers, and the public. Radiation poses radiological risks for people in proximity to diagnostic equipment, e.g., scanners and X-ray machines, and other instruments [1,2]. Protection against X-rays involves both source shielding (usually supplied by the manufacturer) and structural shielding, which involves rooms that contain diagnostic or radiation therapy X-ray machines, sealed-source therapy units, unsealed sources of radiation, or linear accelerators [3]. The new recommendations for shielding design for medical imaging facilities and therapeutic facilities can be found in the National Council on Radiation Protection (NCRP) publication [4]. Shielding design involves primary barriers and/or secondary barriers depending on the facility. For example, in a chest X-ray room, there is scattered radiation from patients undergoing X-rays who become a source of scattered radiation along with the surrounding materials irradiated by the primary beam. The radiation also includes leakage radiation from the tube or source housing. Personal protective equipment may include wearable or nonwearable radiation protection equipment such as aprons, gloves, garments, eyeglasses, thyroid shields, face shields, mobile barriers, etc., depending on the facility and the radiation procedure’s requirements. In a PET facility, the patient is injected with beta-plus emitters and becomes an extended source of annihilation gamma radiation at 0.511 MeV, which is higher than the level usually encountered in diagnostic radiology. This requires greater thicknesses of materials to be used for structural shielding. In all circumstances, the ALARA (as low as reasonably achievable) principle must be rigorously implemented to minimize exposure to radiation through time, distance, and shielding rules. For many years, lead was used widely for shielding gamma rays and X-rays. Due to the toxicity of lead and its heavy weight, there is a rapidly expanding research focus on developing new and safe radiation shielding materials. Lead (Pb) toxicity is an important environmental health issue worldwide, affecting almost all functions in the human body. Impacted organs and systems include the immune and reproductive systems, brain and nervous system, gastrointestinal tract, liver, endocrine, kidneys, and skin. Lead is poorly excreted by the human body, leading to both chronic and acute disorders. Different pathways permit lead to enter the human body. These include the ingestion or inhalation of lead particulates from lead dust, food, and soil, and contact with lead products [5,6]. Studies have shown that the effects of lead toxicity on the human body are devastating and may include developmental problems in behavior, growth, learning, hearing, and speech [7]. The number of deaths due to lead exposure reached 902,000 in 2019 and the number of disability-adjusted life years (DALYs) reached 21.7 million worldwide. Lead accounts for 1.5% (900,000) of deaths annually in the world, as recorded by the Institute for Health Metrics and Evaluation (IHME) [8]. With the emergence of nanotechnology, the development of new lead-free composite materials that are safe, environmentally friendly, lightweight, and reliable for radiation shielding is expanding rapidly in different radiation-related sectors, such as healthcare (radiotherapy and medical imaging), nuclear power plants, industry, and others. This has necessitated the development of new fabrication and characterization techniques [9,10]. In this respect, polymer microcomposites and nanocomposites are increasingly being applied in different applications including X-ray- and gamma-ray-shielding composites [11,12]. These composite materials present numerous advantages. By choosing different matrices and different fillers, it is possible to produce composite materials that are mechanically stable, solid, and flexible. Their physical and chemical properties can be modified according to requirements by varying these parameters. Various polymers are commonly used for the fabrication of polymer matrices reinforced with metallic composites or nanocomposites as fillers [13]. High-Z metals (tungsten and bismuth, for example) are used as alternatives to lead because they are safe and have low toxicity [14]. Other lower-Z inorganic fillers have also been used by different authors. The particle size of the filler has been found to positively affect the attenuation coefficient of X-rays and gamma rays. El-Khatib et al. concluded that high-density polyethylene (HDPE)/CdO nanocomposites had greater mass attenuation coefficients compared with CdO/HDPE microcomposites at the same filler loadings, but to different extents depending on the energy used and the weight percentage of filler [15]. Nanofillers permit a more uniform dispersion with less agglomeration into the polymer matrix. Similarly, Li Ran et al. concluded in their work on epoxy resin filled with nano- and micro-Ga_2_O_3_ that the enhancement of shielding properties was stronger at low photon energies within the energy range of 31 to 356 keV [16]. However, Azman et al. concluded in their work on X-ray transmission using WO_3_ nano- and microfillers that the effect of particle size was negligible at the higher X-ray tube voltages used in the radiography unit (40–120 kV) compared to those used in the mammography unit (25–35 kV) [17]. Abu Saleem et al. attributed the enhancement of shielding properties when using nanofillers to their more uniform dispersion within the polymer matrix and an increased surface-to-volume (S/V) ratio, leading to increased electron density and photon interactions compared to microfillers [18]. Different methods for polymer composite synthesis are used, such as the melt-mixing method, compression molding technique, ultraviolet (UV) curing, and others [10]. Different characterization methods have been used to analyze the prepared samples. Different experimental techniques using different detectors have also been used to measure the mass attenuation coefficients within energy ranges of interest.

Kucuk et al. experimentally measured the mass attenuation coefficients at different energies (from 60 to 1332 keV) for some polymers, namely PMMA, polyamide (PA-6), polystyrene (PS), low-density polyethylene (LDPE), polyethylene (PE), and polypropylene (PP). They concluded that PMMA and PA-6 had better photon absorption characteristics compared to other investigated polymers. In addition, both showed better thermal and mechanical properties than others did [19]. Besides that, PMMA is easy to manipulate, has low cost and is biocompatible, which is the reason for its wide use in dentistry [20]. Consequently, in the present work, we have chosen PMMA as the matrix for Bi_2_O_3_ nanofillers. Alshahri et al. prepared LDPE/ Bi_2_O_3_ nanocomposites for shielding X-rays at 5%, 10%, and 15% filler weight, which were investigated at the following X-ray energies: 47.9, 100.0, 118.0, 165.0, 207.0, and 248.0 keV using an X-ray source and an ionization chamber detector. In addition, their mechanical and thermal properties were studied accordingly. They found that the LDPE+Bi_2_O_3_ (15%) composite had better shielding properties and was able to block 80% of X-rays at 47.9 keV [21]. Ambika et al. studied the role of Bi_2_O_3_ as a filler on the gamma shielding performance of an unsaturated polyester (UP)-based polymer composite at 80, 356, 662, 1173, and 1332 keV using a NaI (Tl) detector. Their results revealed that the linear attenuation coefficient increased when filler wt.% increased and decreased when X-ray energy increased [22]. In similar work, they prepared an isophtalmic-Bi_2_O_3_ polymer composite to study their shielding properties at the Cs-137 gamma ray (662 keV) energy using a NaI (Tl) scintillation detector along with their mechanical, thermal, and electrical properties. The filler weight percentages were 0, 10, 20, 30, 40, 50, and 60%. They concluded that the measured mass attenuation coefficient at 662 keV increases with increasing Bi_2_O_3_ content [23]. Mehara et al. fabricated a Polycarbonate (PC)/bismuth oxide nanocomposite for low-energy gamma ray shielding at 59, 122, 140, and 356 keV energies using a CsI (Tl) scintillator detector. The filler weight percentages were 0, 10, 20, 30, 40, and 50%. They found out that increasing the Bi_2_O_3_ filler concentration significantly increases the attenuation coefficient [24]. Cao et al. investigated the gamma shielding properties of PMMA/Bi_2_O_3_ microcomposites fabricated by the fast ultraviolet (UV) curing method at energies of 88, 122, 356, 662, 1173, and 1333 keV using a NaI (Tl) scintillation detector. The filler weight percentages were 0, 15.6, 24.6, 34.3, and 44.0%. They showed that these composites have enhanced gamma shielding ability compared with pure PMMA for gamma energies up to 1000 keV. In addition, their mechanical hardness improved with higher Bi_2_O_3_ filler loading, leading to excellent mechanical and shielding properties [25]. Mahmoud et al. studied the shielding properties of prepared silicon rubber (SR)/Bi_2_O_3_ composites at energies of 60, 81, 356, 662, 1173, and 1333 keV using a NaI (Tl) scintillation detector. The filler weight percentages were 0, 5, 10, 20, and 30%. They concluded that SR nanocomposites had better attenuation properties than their micro counterparts at all discussed energies. In addition, the mechanical properties of synthesized composites were studied as well [26]. Pavlenko et al. prepared polyimide/Bi2O3 composites and studied their surface, physical-mechanical, and shielding properties [27]. They concluded that that the composites exhibited high gamma radiation protection in the 0.1–1 MeV energy range. Other authors used different fillers than Bi_2_O_3_ and different matrices. Chaitali et al. presented a review about different polymeric composite materials for radiation shielding, showing the obtained results for the mass attenuation coefficient at 59.53, 80.99, 356.01, 661.66, and 1173.25 keV, wherever applicable [28]. They emphasized that polymers are promising due to their mechanical, electrical, thermal, and multifunctional properties. The synthesis of polymer composite materials for radiation protection was reviewed, with a focus on the role of the nanofillers. In addition, they discussed the effectiveness of polymeric materials for the absorption of fast neutrons and discussed the recycling of polymers into composites. They concluded that polymer materials are promising candidates for mixed neutron–gamma-rays shielding [28].

Tungsten, cadmium, bismuth, and gallium oxides and other elements were used in nanocomposites. We have opted for bismuth element (Z = 83) because it is a high-Z element and has similar shielding properties to its neighbor element lead (Z = 82) for a wide range of photon energies of interest as revealed in the NIST database [29]. Tungsten (Z = 74) also exhibits very good shielding properties due to its huge density (19.3 g/cm^3^) and relatively high Z. Its HVL at Co-60 gamma energies (7.9 mm) is smaller than that of lead (12.5 mm), which has a density of 11.3 g/cm^3^. It is the second-densest element after Osmium. It is safe, but unfortunately, it is costly. Cadmium element (Z = 48) and gallium element (Z = 31) have lower attenuation capabilities at low energy, where the photoelectric effect is dominant, due to their smaller atomic number. Both CdO and Ga_2_O_3_ are used as lightweight shielding as alternatives to lead. However, cadmium is classified as a toxic substance by the agency for toxic substances and disease registry under Id CAS ID#: 7440-43-9. Gallium is not harmful but has a high commodity price due to its wide use in the electronic industry. As an element, bismuth is nontoxic. However, its excessive consumption, usually from medicinal use, may lead to toxic exposure [30].

In fact, there are a number of studies using bismuth in different areas of science and technology due to its excellent electrical and optical properties. It has a wide band gap, high dielectric permittivity, good photoconductivity, and high refractive index. In the field of shielding, several studies involved Bi_2_O_3_ as micro- or nanofillers for different polymeric matrices such as HDPE, PI, epoxy, polyester resin, poly-dimethyl siloxane (PDMS), and polycarbonate and silicon resin, targeting different energy ranges (from low energy X-rays up to 1332 keV photons) [31]. To the best of our knowledge, based on the available literature, only one work involved Bi_2_O_3_-PMMA microcomposites manufactured by the UV curing method to study their shielding properties for similar photon energy ranges as those of interest to us [25]. In our case, the manufacturing method and filler size differed from Cao’s work, in which the average size of Bi_2_O_3_ particles was 2–5 μm. Therefore, our present work is the first of its kind about Bi_2_O_3_-PMMA nanocomposites manufactured by the melt-mixing method at 210–220 °C.

In the present study, the morphology of PMMA-Bi_2_O_3_ polymer nanocomposites prepared by the melt-mixing method was studied by scanning electron microscope (SEM). Their crystalline structure was investigated by X-ray diffraction (XRD). Thermogravimetric analysis (TGA) was performed to investigate the thermal stability of prepared nanocomposites at 0, 10, 20, 30, and 40 weight percentages. Gamma spectrometry with a NaI (Tl) scintillation detector has been used to examine gamma radiation shielding properties. A wide range of energies from 60 keV to 1332 keV has been investigated as they are applicable to the medical field (such as diagnostic radiology, medical imaging, and radiation therapy) and to other radiation-related fields.

## 2. Materials and Methods

### 2.1. Fabrication of Polymer Nanocomposites

**Figure 1:**
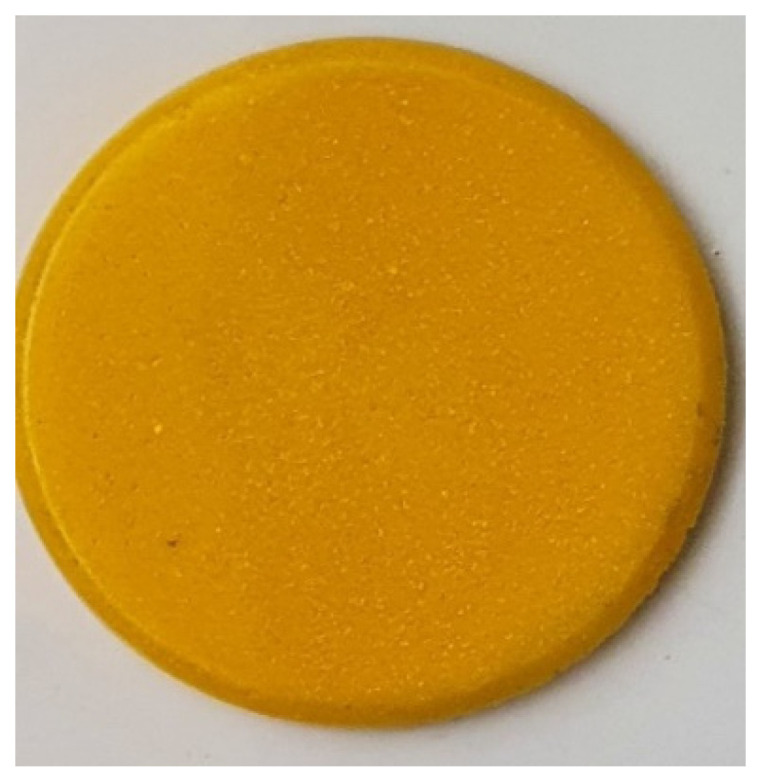
A specimen of synthesized PMMA nanocomposite at 30% loading with Bi_2_O_3_ filler.

Bismuth (III) oxide (Bi_2_O_3_) nanopowder was purchased from ASNA Co. for Advanced Technologies Ltd. (Bismuth (III) oxide (provided by Sigma Aldrish, Darmstadt, Germany) with particle size 90–210 nm. Bismuth oxide (a direct bandgap semiconductor) was chosen because of its high gamma attenuation capabilities similar to toxic lead. It is a high-Z element (Z = 83) with a high density of 8900 kg/m^3^ and high melting point of 817 °C. General-purpose PMMA material was purchased from local supplier, which was transformed into fine powder using an ultra-high-speed electrical grinder. Bismuth oxide nanopowder (Bi_2_O_3_) was mixed with the PMMA powder at 10%, 20%, 30%, and 40% weight percentages. A very sensitive balance (CITIZEN SCALE CX 301, Mumbai, India, d = 0.1 mg) was used for weighing both powders accurately. Before heating in oven, the two powders were very well mixed with a blender throughout. The polymer nanocomposites were prepared by melt-mixing method. The melted composite at temperature ~210 °C was once more gently mixed mechanically and heated again at 220 °C to reduce eventual pores formation as much as possible. After that, they were cold pressed immediately into cylinder molds with 3 cm diameter, 3 mm and 5 mm thicknesses. Finally, the molds were left under press until cooled to room temperature. Figure 1 shows a specimen of prepared nanocomposite at 30% loading with Bi_2_O_3_ filler. The density of prepared samples was measured by Archimedes’ method using distilled water. Finally, it is worth mentioning that all prepared nanocomposites showed a high mechanical strength when we used high-speed mini electric grinder and metal cutting saw to cut them. Coolant water was necessary to avoid melting. The acrylic matrix of prepared nanocomposites gave them shatter resistance and strength. These very good shielding and mechanical properties permit their use as effective shielding barriers in diagnostic and nuclear medicine compartments such as computed tomography (CT), X-ray, angiography, fluoroscopy, mammography, and radiotherapy. They can be used in mobile X-ray barriers to provide protection from scattered radiation or as gonad shields. The prepared composites sheets can be cut (reshaped) to fit special procedures such as in interventional radiology. For example, thyroid shields are recommended during diagnostic medical and dental radiology [32]. They may be used to replace lead-lined boxes in the case of an X-ray tube used by a radiologist to irradiate small organisms. They can be used as shielding around a room where radiation sources or radiation therapy is used for patient diagnosis procedures [3]. However, in order to prepare wearable personnel protective equipment such as aprons and gloves, or manufacture X-ray curtains, a flexible matrix (such as vinyl) is required instead of PMMA.

Quality control of shielding barriers should be performed after long-time exposure to radiation in order to check for possible radiation degradation, especially those in high radiation field facilities, such as LINAC (Linear Accelerator) and radiotherapy. As a matter of fact, White et al. found that most polymers can tolerate radiation in the range of 10 to 100 Gy with no degradation of mechanical properties. For much higher doses (1 kGy to 10 kGy), many polymers can accommodate it with little to no consequence [33]. However, in nuclear applications, a degradation of the polymer chain was noticed due to long-term gamma exposure. The products become useless due to the degradation of their mechanical and thermal properties [34]. This phenomenon is related to radiation-induced aging.

### 2.2. Characterization of Fabricated Polymer Nanocomposites

#### 2.2.1. Scanning Electron Microscopy

The surface morphology of the synthesized PMMA-Bi_2_O_3_ nanocomposites as well as pristine PMMA were performed at the center of nano Technology (CNT) at King Abdulaziz University (KAU), Jeddah, Saudi Arabia, using scanning electron microscope (SEM) with model number LYRA3 TESCAN. The binding and dispersion of bismuth oxide filler within the matrix were obtained for different filler percentages.

#### 2.2.2. X-ray Diffraction

The crystalline structure was investigated at the center of nano technology at KAU using X-ray diffraction (XRD) coupled with Ultima-IV diffractometer from Rigaku, Japan. The changes in diffraction patterns that occurred due to bismuth oxide filling the PMMA matrix were recorded for different filler percentages. The characteristic Cu-Kα X-ray with wavelength λ = 0.15406 nm was used for this purpose at room temperature. The average crystallites sizes D were estimated using Scherrer’s formula from the obtained XRD patterns at different Bi_2_O_3_ loadings. Scherrer’s formula for crystallite size is given by the following [21]:

(1) $$D\left(\mathrm{nm}\right)=\frac{K\lambda }{\beta \;\cos\theta }\;$$

where β is the FWHM in radians of XRD diffraction peak (centered at angle θ) and K = 0.94 is the crystallite shape factor.

#### 2.2.3. TGA Analysis

Thermogravimetric analysis was carried out using METTLER TOLEDO, Columbus, Ohio, USA TGA/DSC 1 STAR System. The thermal stability of synthesized nanocomposites along with pristine PMMA was analyzed by recording various thermograms from 25 °C up to 600 °C at a heating rate of 10 °C/min.

### 2.3. Gamma-Ray Shielding Properties of Fabricated Polymer Nanocomposites

#### 2.3.1. Theoretical Overview

The intensity of a transmitted beam of X or gamma radiation (for narrow beam geometry) through a shielding material of linear thickness x is given by the Beer–Lambert law [35]:

(2) $$I={\;I}_{0}e^{-\mu .x}$$

where μ (cm^−1^) is the linear attenuation coefficient of the shielding material. I_0_ and I are the incident and transmitted photon intensities, respectively. The lμ coefficient is extracted by fitting the experimental data performed for different thicknesses with Equation (2). The mass attenuation coefficient, which is independent of the density (ρ) of the material, is given by

(3) μm=μρ

In terms of μ_m_ (cm^2^ g^−1^), Equation (2) is rewritten as

(4) $$I={\;I}_{0}e^{-\mu_{m}.d}$$

where d [(d = x.ρ) (g cm^−2^)] is the mass per unit area (i.e., the mass thickness of the sample). The contribution to the attenuation is mainly from photoelectric effect, Compton scattering, and pair production at energies greater than the threshold energy of 1.022 MeV. The cross section of each interaction and its contribution to the total attenuation coefficient depends on the photon energies, the atomic numbers (Z) (of constituents of mixtures or compounds), and the density of the material. The linear attenuation coefficient decreases with increasing photon energy except at K-edges where an abrupt increase occurs in the photoelectric cross section. It is worth noting that the K-absorption edge of bismuth (at 90.5 keV with Z = 83) is slightly above that of lead (at 88.0 keV with Z = 82). The photoelectric effect has an absorption cross section that strongly depends on E and Z number (of the absorbing medium) as σ_pe_~Z^4^/E^3.5^ [35]. Therefore, it is dominant at low energy and higher-Z materials, which makes high-Z elements such as lead and bismuth better shielding materials against X-rays. Compton scattering is the dominant effect for medium-energy photons and its probability depends on the electron density in the absorbing material. Pair production has a threshold energy of 1022 keV (electron–positron pair production in the field of nucleus) and a cross section proportional to Z^2^. Therefore, it is insignificant in our current study of 60 keV–1332 keV energy range.

#### 2.3.2. Experimental Determination of Gamma Rays Attenuation Coefficients

**Figure 2:**
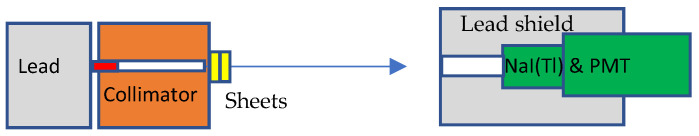
Schematic diagram for the experimental setup under narrow beam geometry. Radioactive source in red inside collimator hole in white.

The measurement of mass attenuation coefficients was performed using a complete gamma-ray spectrometry system with personal computer (PC)-based multichannel analyzer (MCA). A 2.5″ × 2.5″ NaI (Tl) scintillation detector with 7.2% resolution at the Cesium-137 line (662 keV) was used in a narrow beam (good) geometry set up with a lead collimator around the source and a lead shield around the detector entrance to eliminate backscattered radiation as shown in Figure 2. The measurement spanned a wide range of energies that are of interest to medical applications for diagnosis and therapy using different radioisotopes: ^241^Am (59 keV), ^57^Co (122.2 keV), ^226^Ra (242.0 keV), ^133^Ba (80.99 and 356.02 keV), ^137^Cs (661.6 keV), and ^60^Co (1173.2, and 1332.5 keV). The distance between the detector entrance and radioactive source was ~40 cm. In the narrow beam transmission experiment, the incident and transmitted intensities were determined by the net area under relevant photo peaks, i.e., region of interest (ROI). The obtained results were then fitted to Beer–Lambert’s law to extract the linear and mass attenuation coefficients. Figure 2 shows the experimental setup. During the gamma attenuation measurement, the thickness of each sample was recorded separately for accuracy using a micrometer.

## 3. Results

### 3.1. Characterization

#### 3.1.1. Scanning Electron Microscopy

**Figure 3:**
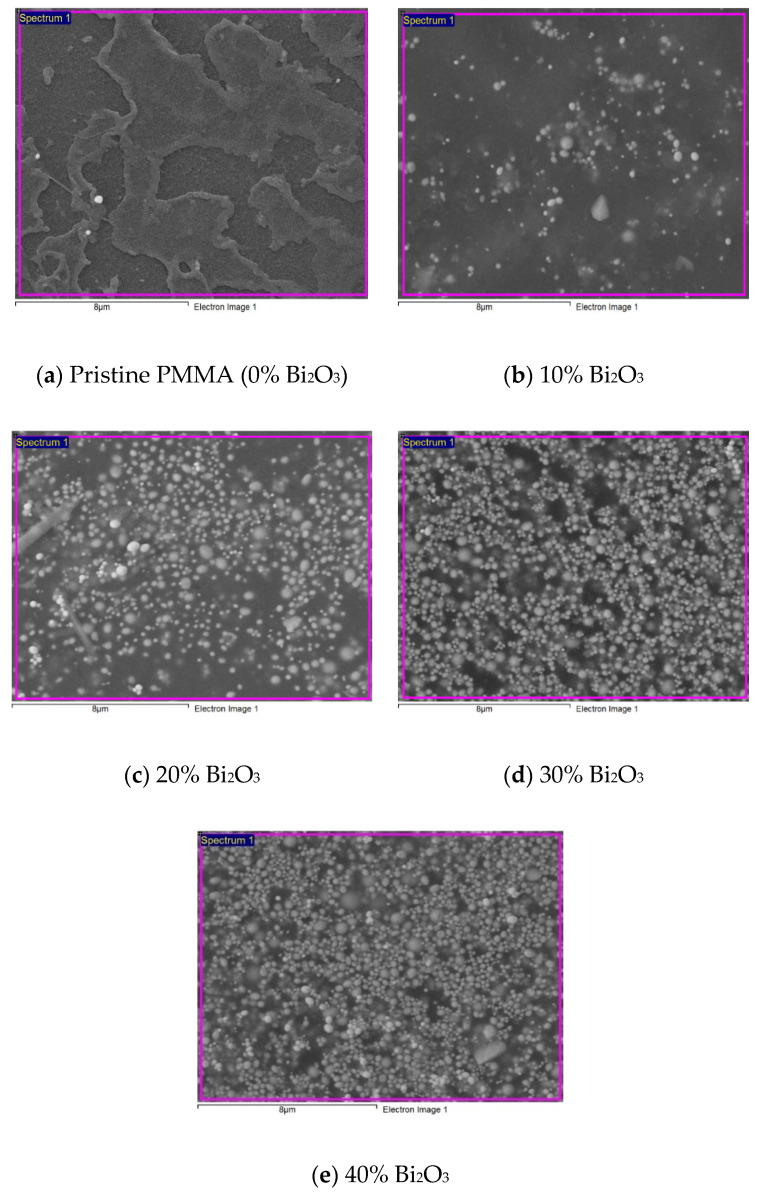
SEM images of pristine PMMA and synthesized PMMA-Bi_2_O_3_ nanocomposites at different filler loadings: (**a**) 0%; (**b**) 10%; (**c**) 20%; (**d**) 30%; (**e**) 40%.

Figure 3 displays the SEM images of pristine PMMA and synthesized PMMA-Bi_2_O_3_ nanocomposites for 0%, 10%, 20%, 30%, and 40% filler loadings. A good dispersion of the Bi_2_O_3_ nanofiller within the PMMA matrix with increasing concentrations by increasing the filler weight percentage was achieved. A close look at the SEM images shows that the claimed particle size 90 to 210 nm of the purchased nanopowder is indeed verified overall with the exception of a few agglomerations (with diameters ~400 to 600 nm). There was no contamination in all samples, as shown in the Energy-Dispersive Spectroscopy (EDS) images provided in the Supplementary Materials of SEM-EDS images in Figures S1–S5.

#### 3.1.2. X-ray Diffraction Analysis

**Figure 4:**
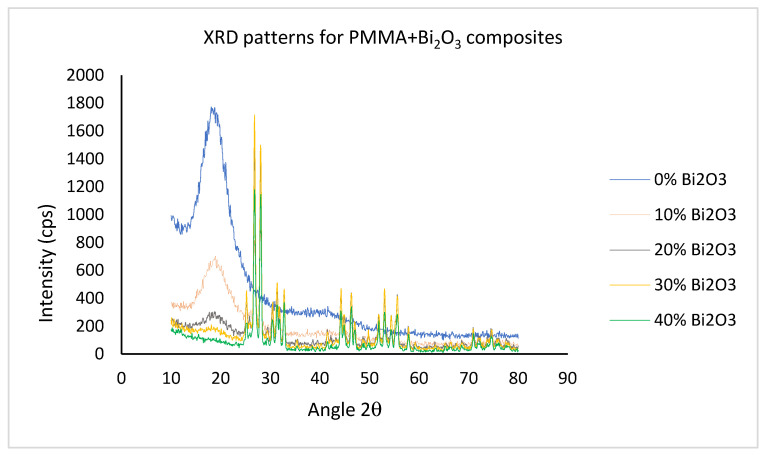
XRD spectra of pristine PMMA and synthesized PMMA-Bi_2_O_3_ nanocomposites for 0%, 10%, 20%, 30%, and 40% filler loadings.

Figure 4 shows the XRD spectra of pristine PMMA and synthesized PMMA-Bi_2_O_3_ nanocomposites for 0%, 10%, 20%, 30%, and 40% filler loadings. The XRD pattern of the used matrix, i.e., PMMA sample (0% Bi_2_O_3_ blue plot), indicates its amorphous nature with a broad hump diffraction peak centered at 2θ = 18.84°. A similar value of 2θ = 19.12° was reported by Abasi et al. [36]. Filling the PMMA matrix with Bi_2_O_3_ nanoparticles at different loading % led to XRD patterns with well-defined peaks with the prominent ones at 25.35°, 26.82°, 28.02°, 30.41°, 31.33°, 31.87°, 32.76°, 44.27°, 44.8°, 46.30°, 46.4°, 46.99°, 46.99°, 51.9°, 53.02°, 54.5°, and 55.54°, and others with less intensity at higher diffraction angles. As loading with the Bi_2_O_3_ filler increased, the broad hump attributed to pristine PMMA diminished considerably. At 40% loading, the PMMA hump almost disappeared completely.

The obtained complex spectra may indicate the presence of two phases: tetragonal β-Bi_2_O_3_ and monoclinic α-Bi_2_O_3_ with some interferences. Different crystallographic cards were assigned to Bi_2_O_3_ by different authors [21,23,24,37,38] according to their composites’ spectra. However, the most intense peak of β-Bi_2_O_3_ that is well defined at 2θ = 28.02° in this study is in agreement with reported values of 27.94°, 27.80°, and 27.92°, respectively, by [21,37,38]. It is assigned to the (201) reflection plane of the tetragonal Bi_2_O_3_ crystal structure [23]. In addition, the most intense peak of the monoclinic structure α-Bi_2_O_3_ is well defined at 2θ = 26.83° in this study and is assigned to the (111) reflection plane. It is in agreement with the reported value of 26.92 by Hou et al., who synthesized α-β phase heterojunction on Bi_2_O_3_ nanowires and therefore obtained XRD spectra comprising both phases [37].

They reported that when the hydrothermal temperature was increased above 150°, the α-phase appeared in addition to the β-phase. At higher temperatures, 180–240°, the monoclinic α-Bi_2_O_3_ (PDF NO. 4-294) dominated over the tetragonal β-Bi_2_O_3_ phase (PDF NO. 18-244). It is worth mentioning that there are six polymorphic forms of Bi_2_O_3_ reported by researchers, which include monoclinic (α-Bi_2_O_3_), tetragonal (β-Bi_2_O_3_), cubic body-centered (γ-Bi_2_O_3_), cubic face-centered (δ-Bi_2_O_3_), orthorhombic (ε-Bi_2_O_3_), and triclinic (ω-Bi_2_O_3_) phases [39]. Table 1 displays the average crystallites sizes D that were estimated using Scherrer’s formula from the obtained XRD patterns at different Bi_2_O_3_ loadings. The average crystallite size derived from the two strongest peaks (with “similar” intensities) was 26 nm, ranging from 24 nm to 27 nm.

The presence of both tetragonal β-Bi_2_O_3_ and monoclinic α-Bi_2_O_3_ phases and their interferences may have affected the computed values to some extent. The separated XRD spectra for different loadings are given in the Supplementary Materials in Figure S6a–e for clarity.

#### 3.1.3. TGA Analysis

**Figure 5:**
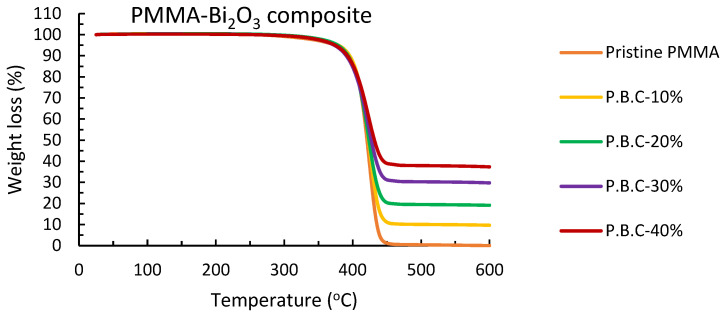
TGA thermograms of pristine PMMA and synthesized PMMA-Bi_2_O_3_ nanocomposites (P.B.C) for 0%, 10%, 20%, 30%, and 40% filler loadings.

Thermogravimetric analysis was performed between 25 and 600 °C to investigate the thermal stability of synthesized nanocomposites. Figure 5 shows the TGA thermograms of pristine PMMA and synthesized PMMA-Bi_2_O_3_ nanocomposites for different filler loadings. Table 2 presents their analysis. The residual weight percentages at 600 °C are 9.68, 19.12, 29.75, and 37.36 in accordance with the 10%, 20%, 30%, and 40% presumed initial Bi_2_O_3_ loadings, respectively, within experimental errors. This may indicate a good uniform dispersion of Bi_2_O_3_ nanoparticles into the polymer matrix. In addition, a gradual shift in the extrapolated onset temperature toward lower temperature values is noticed, as shown in Table 2. The extrapolated onset temperature is extracted according to the International Organization for Standardization (ISO) standard ISO 11358-1 [40]. This shift is related to a decrease in the rate of mass loss (degradation) as loading weight percentage is increased, as shown in Figure 5. This indicates that the thermal stability of polymers (PMMA in our case) increases when their loading with inorganic filler (Bi_2_O_3_ in our case) is increased, as reported by other authors [21,24]. High peak temperatures are extracted from corresponding TGA curves and are given in Table 2 as well.

### 3.2. Gamma Ray Attenuation Coefficients Analysis

**Figure 6:**
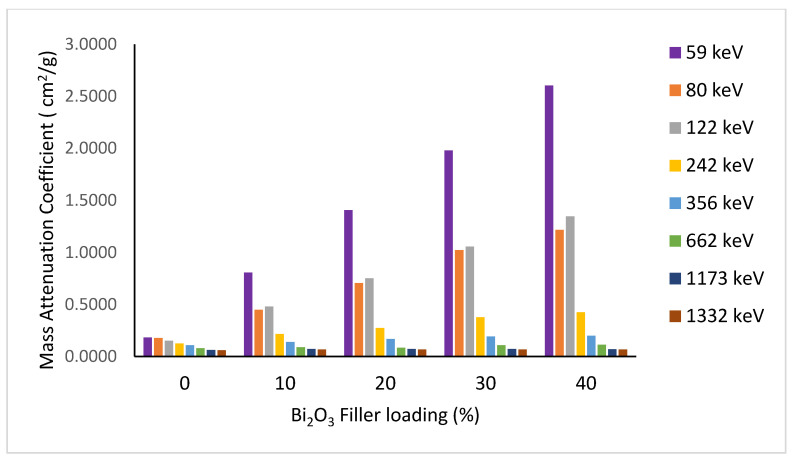
Experimental results for the mass attenuation coefficient variation with Bi_2_O_3_ loadings at different energies.

Shielding properties are best described by the mass attenuation coefficient of the absorbing material. The incorporation of a high-Z element (Bi_2_O_3_) into the PMMA polymer matrix considerably enhanced the shielding properties of the composite. Figure 6 shows the dependence of the mass attenuation coefficient on the filler loading at different energies. Better attenuation properties are obtained with increasing filler weight percentage, especially at low and medium energies up to the measured 662 keV.

**Figure 7:**
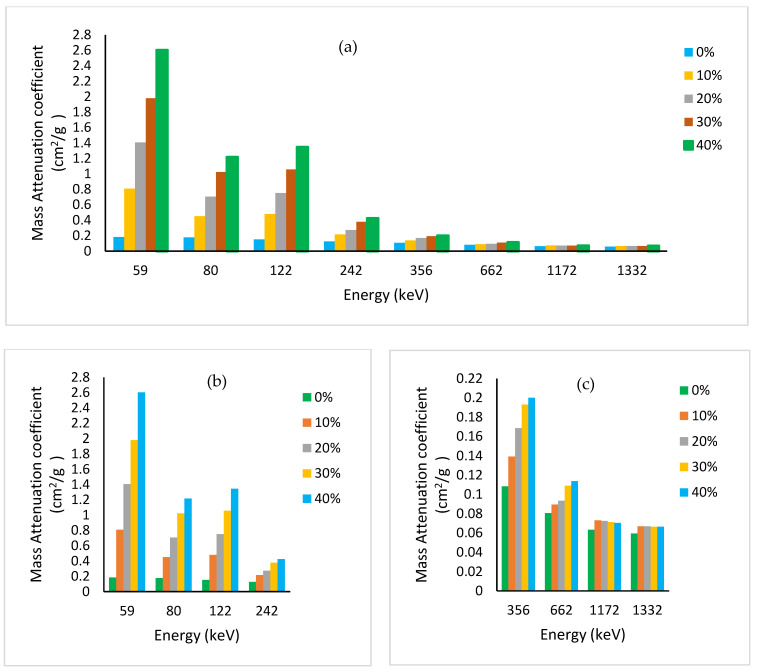
Experimental results for the mass absorption coefficient variation with energy at different Bi_2_O_3_ loadings: (**a**) all measured energies; (**b**) low and medium energies; (**c**) high energies.

At 40% loading of Bi_2_O_3,_ the mass attenuation coefficient for the cesium gamma line at 662 keV has reached the corresponding value for the toxic heavy element lead (μm = 0.104 cm^2^/g). At high energy (~1 MeV), the Compton effect dominates with μ_m_ becoming almost constant for all elements independent of the atomic number Z of the absorbing material, except hydrogen (Z/A = 1). Pair production is insignificant in our measured energy range of interest, 60 to 1332 keV. Figure 7a shows the measured mass attenuation coefficient over the whole studied energy range for different filler loadings. For clarity, the low–medium and high energy portions are drawn separately in Figure 7b,c, respectively.

**Figure 8:**
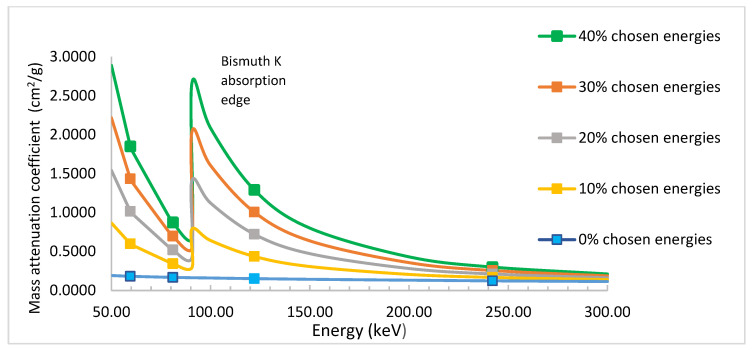
XCOM theoretical simulation for the mass absorption for low energies showing Bismuth K-absorption edge effect.

It is worth mentioning that our measured values for pristine PMMA are similar to those obtained by Manjunatha, within the evaluated error of 2–3% using Equation (12) in his paper [41]. He used a similar experimental setup as ours, with a similar scintillation detector to measure the mass attenuation coefficient for PMMA and Kapton at the following energies: 84, 122, 145, 279, 320, 392, 511, 662, 1170, 1274, and 1332 keV. For our prepared PMMA-Bi_2_O_3_ nanocomposites, the mass attenuation coefficient is found to decrease with increasing photon energy except near the K-absorption edge of bismuth at 90.5 keV, where an abrupt increase occurs due to the photoelectric cross section. This is well demonstrated by the increase in the mass attenuation coefficient at 122 keV energy above that of the nearby 81 keV measured energy. For the sake of clarity, we deliberately performed the XCOM simulation based on the NIST database for the PMMA-Bi_2_O_3_ mixture for 0%, 10%, 20%, 30%, and 40% filler loadings [29]. The plot has been restricted to the low-energy portion, 50–300 keV, for clarity, as shown in Figure 8. The dots represent the chosen energies in this range for the respective curves (60, 80, 122, and 242 keV). It is worth noting that for much lower-Z nanofillers, this behavior is not observed because of their lower K-absorption edge. For example, Al-Khatib et al. used CdO nanofillers and found that the mass attenuation coefficients at 80 keV were higher than at 122 keV [15]. The reason is that cadmium has a K-absorption edge at a much lower energy (26.7 keV) than bismuth at 90.5 keV.

**Figure 9:**
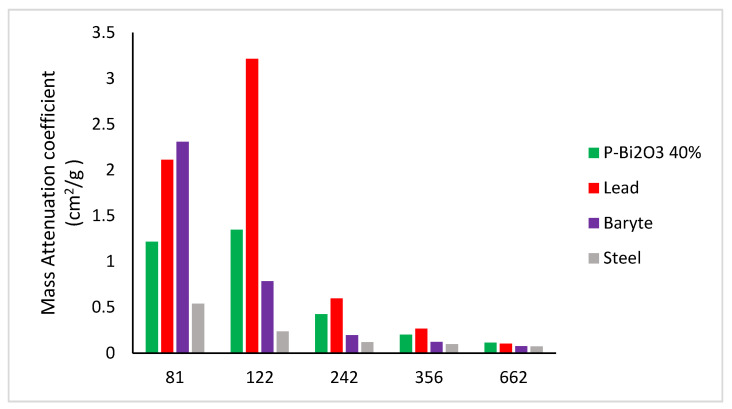
Comparison of the mass attenuation coefficients of synthesized PMMA-Bi_2_O_3_ nanocomposites at 40% loading with their corresponding ones for lead, steel, and baryte.

However, at higher energies (1173 and 1332 keV), the Compton effect is dominant and the variations in the mass attenuation coefficient become minor. Finally, the mass attenuation coefficients obtained for synthesized PMMA-Bi_2_O_3_ nanocomposites for the energy range (81–662 keV) are compared to their corresponding ones (XCOM-derived) for lead, steel, and baryte as shown in Figure 9. At 30% loading of Bi_2_O_3_, the mass attenuation coefficient for the cesium gamma line at 662 keV has reached the corresponding value for the toxic heavy element lead (0.104 g/cm^2^) and has surpassed it at 40% loading by 10%.

For the lowest measured energy at 59 keV, the mass attenuation coefficient is highest for barite with a value of 5.171 cm^2^/g compared to lead, steel, and PMMA-Bi_2_O_3_ at 40% loading, with values of 4.738, 1.164, and 2.603 cm^2^/g, respectively.

**Figure 10:**
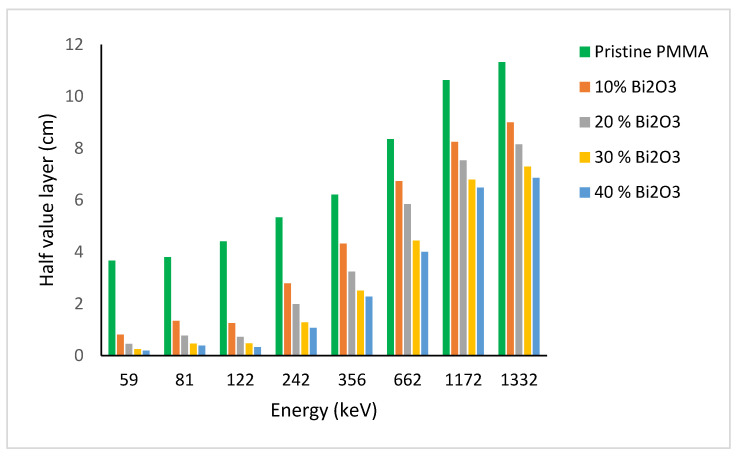
Experimental results for HVL (half value layer) variation with energy at different Bi_2_O_3_ loadings.

In view of the toxicity and heaviness of lead, we consider PMMA-Bi_2_O_3_ nanocomposites to be a very effective and efficient alternative for radiation shielding in the health sector for the whole studied energy range (<59 keV to 1332 keV). It is worth noting that theoretical XCOM-based simulations for computing the mass absorption coefficient for different mixtures and compounds do not take into account the filler size, density, and dispersion uniformity in the matrix. MNCPX simulations performed by Tekin et al. on concrete filled with WO_3_ nanoparticles showed that they have better shielding properties than their micro counterparts for the same fillers and same loadings [42]. The experimental results for the half value layer (HVL = ln2/μ) variation with energy at different Bi_2_O_3_ loadings, for all measured energies, are shown in Figure 10.

HVL is defined as the thickness of shielding material required to attenuate the incident photon intensity by half (i.e., 50% reduction). Figure 10 shows that the HVLs for pure PMMA are the largest at all energies, indicating their poor shielding ability. On the contrary, HVL is decreasing considerably with increasing filler loadings in the matrix, especially at low energies. As the energy is increased, photons become more penetrating, leading to an increase in HVL, especially at 1332 keV. For the Cs-137 gamma line at 662 keV, HVL is equal to 4.0 cm and 4.4 cm for 40% and 30% loadings, respectively, compared to 0.7 cm for lead. It is worth noting that at 122 keV, HVL is a bit smaller than at 81 eV due to the K-absorption edge of bismuth as explained before. The 59 keV is the most attenuated due to the higher photoelectric cross section, which varies as σ_pe_~Z^4^/E^3.5^ [22]. In addition, Bi_2_O_3_ polymeric composites are very efficient at lower energies as reported elsewhere [21,27].

To gain more insight on the importance of the acquired results, the thickness required for an attenuation of 75% or 90% of the incident photons is computed for the low-energy portion due its wide use in shielding as derived from Equation (2) [35]:

(5) $$x=-\frac{1}{\mu }\mathrm{Ln}\left(\frac{I}{I_{o}}\right)$$

**Figure 11:**
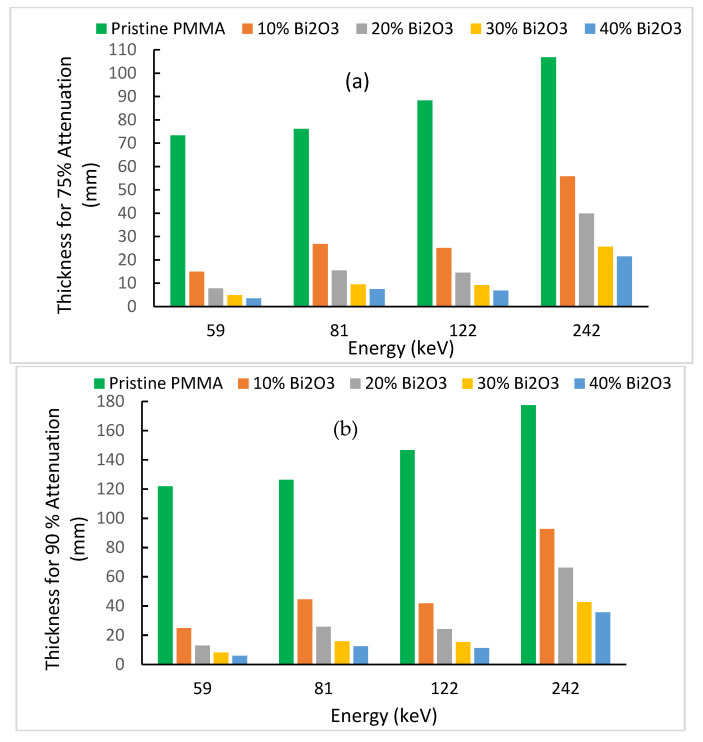
Computed thickness of prepared nanocomposites for (**a**) 75% attenuation; (**b**) 90% attenuation.

The results are plotted in Figure 11a,b for photon energies of 59, 81, 122, and 242 keV. According to the narrow X ray spectrum qualities used (adopted from ISO 4037 [21,43]), the tube potentials (kVp) of 60, 100, 150, and 300 kV have effective energies of 47.9, 83.3, 118, and 248, respectively. The last three tube effective energies are very close to our used radioisotopes gamma energies of 81, 122, and 242, respectively.

Figure 11 shows clearly that pristine PMMA (polymer) has limited radiation shielding ability at these energies. It requires unrealistic thicknesses of 122 mm or 147 mm to shield 90% of photons, with 59 keV or 122 keV, respectively. As the loading of the polymer with Bi2O3 nanoparticles increases, a smaller shielding thickness is required for a specific energy. As energy increases in the previous range, a larger thickness is required in general (except near the K-absorption of bismuth). Let us consider, for example, the Am-241 gamma line at 59.5 keV, where much of the scattered radiation falls, depending on the radiology facility used. At 40% loading, only thicknesses of 3.5 mm or 5.8 mm are required for 75% or 90% attenuations, respectively. This is a splendid result for fully safe shielding barriers of all kinds (manufactured at 40% nanofiller loading, for example).

Furthermore, let us consider, for example, the Co-57 gamma line at 122 keV, which corresponds approximately to the effective energy of a computed tomography (CT) X-ray tube at 150 kV. Thicknesses of 6.8 mm or 11.3 mm are required for 75% or 90% attenuations, respectively. Again, this is a very good result for such a relatively high X-ray tube voltage and attenuation percentage. It is worth noting that usually sheets of shielding barriers are manufactured with specified thicknesses. They can be superposed to fulfill higher thickness requirements for different diagnostic and therapeutic facilities.

Finally, the previous analysis can be carried out equivalently in terms of the radiation protection efficiency (RPE) defined as follows [28]:

(6) $$\mathrm{RPE}=\left(1-\frac{I}{I_{o}}\right)\times 100\;\left(\%\right)$$

Using Equation (2), we obtain RPE as a function of thickness x and linear attenuation coefficient, which is energy- and shielding-material-dependent:

(7) $$\mathrm{RPE}=\left(1-e^{-\mu x}\right)\times 100\;\left(\%\right)$$

**Figure 12:**
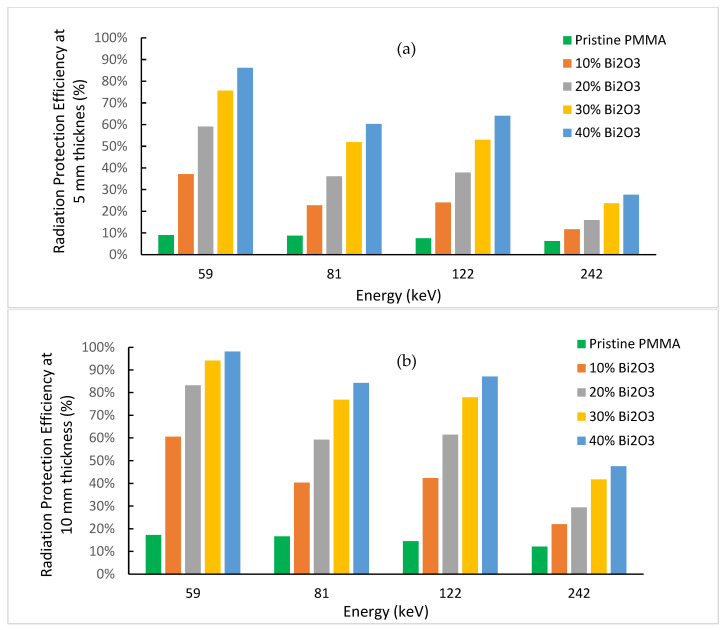
Radiation protection efficiency for prepared nanocomposites at thickness of (**a**) 5 mm; (**b**) 10 mm.

Figure 12 illustrates the variation of RPE at 5 mm and 10 mm thicknesses for prepared PMMA-Bi_2_O_3_ nanocomposites, for the same photon energies at 59, 81, 122, and 242 keV, as before. The pure PMMA showed clearly low shielding abilities. RPE is less than 9% and 17% for 5 mm and 10 mm thicknesses, respectively, in all cases. At 40% loading and a thickness of 5 mm, RPE reached 86% and 64% for 59 keV and 122 keV photons. At a thickness of 10 mm (two superposed sheets of 5 mm for example), RPE jumped to 98% and 87%, respectively. Both results for the 5 mm and 10 mm thicknesses are very promising.

## 4. Conclusions

This study aimed at synthesizing a convenient safe polymeric nanocomposite for gamma radiation shielding in the medical sector principally. PMMA-Bi_2_O_3_ nanocomposites were synthesized by the melt-mixing method and characterized by SEM, XRD, and TGA to study their morphological, structural, and thermal properties. SEM analysis showed a good homogeneity in the dispersion of Bi_2_O_3_ nanoparticles in the PMMA matrix with increased concentrations as filler loadings increased. This was supported by TGA analysis, which showed very smooth thermograms for all synthesized composites ending up with a residual weight at 600 °C in accordance with the initial loadings within experimental errors. Structural analysis with XRD showed the presence of both tetragonal (β-Bi_2_O_3_) and monoclinic (α-Bi_2_O_3_) phases in all nanocomposites synthesized by the melt-mixing method at 220 °C. Gamma spectrometry measurements showed that the mass absorption coefficient (at low and medium energies) increased with filler loadings (and density) and decreased with energy, except near the K-absorption edge of bismuth, where an abrupt increase occurs. At 30% loading of Bi_2_O_3_, the mass attenuation coefficient for the cesium gamma line at 662 keV reached the corresponding value for the toxic heavy element lead and surpassed it at 40% by 10%. Finally, the main objective of the study was achieved successfully. The synthesized PMMA- Bi_2_O_3_ nanocomposites have proven to be very effective, safe, and lightweight shielding materials for a wide range of photon energies from <60 keV to 1332 keV as applied to the medical field (such as diagnostic radiology, medical imaging, and radiation therapy) and to other radiation fields as well.

## Figures and Tables

**Table 1:** Crystallite size for fabricated polymer Bi_2_O_3_ nanocomposites.

Sample	Crystallite Size D (nm)
PMMA + 10% B_i2_O_3_	24(±2)
PMMA + 20% B_i2_O_3_	27(±2)
PMMA + 30% B_i2_O_3_	25(±2)
PMMA + 40% B_i2_O_3_	25(±3)
Average	26(±2)

**Table 2:** TGA thermograms analysis for pristine PMMA and synthesized PMMA-Bi_2_O_3_ nanocomposites (P.B.C) for different filler loadings.

Sample	Bi_2_O_3_ Loading (%)	Extrapolated Onset Temperature (°C)	High Peak Temperature (°C)	Weight (%) at 600 °C
Pure PMMA	0	405.7	422.9	0.03
P.B.C-10%	10	403.5	423.0	9.68
P.B.C-20%	20	399.0	423.1	19.12
P.B.C-30%	30	395.4	423.2	29.75
P.B.C-40%	40	393.1	423.1	37.37

## Data Availability

Data are contained within the article or Supplementary Material.

## Supplementary Materials

The following are available online at https://www.mdpi.com/article/10.3390/polym15092142/s1, Figure S1: SEM-EDS images of pristine PMMA; Figure S2: SEM-EDS images of 10 wt% loading of Bi_2_O_3_; Figure S3: SEM-EDS images of 20 wt% loading of Bi_2_O_3_; Figure S4: SEM-EDS images of 30 wt% loading of Bi_2_O_3_; Figure S5: SEM-EDS images of 40 wt% loading of Bi_2_O_3_; Figure S6: XRD spectra separately for all PMMA-Bi_2_O_3_ Nanocomposites at different loadings: (a) pure PMMA; (b) 10%; (c) 20%; (d) 30%; (e) 40%.
