# Supplementary material for: Study of Prepared Lead-Free Polymer Nanocomposites for X- and Gamma-ray Shielding in Healthcare Applications
Source: Polymers (Basel). 2023 Apr 29;15(9):2142. doi: 10.3390/polym15092142 (PMC10181160; doi:10.3390/polym15092142)
Supplement: Supplementary file 1 [file polymers-15-02142-s001.zip › Supplementary materials cover page.pdf]

Study of Prepared Lead-Free Polymer Nanocomposites for X- and Gamma-ray Shielding in Healthcare Applications

Abdulrhman Hasan Alsaab [1] and Sadek Zeghib *

Physics Department, Faculty of Science, King Abdulaziz University, Jeddah, 21589, Saudi Arabia; aalsaab0031@stu.kau.edu.sa
* Correspondence: abihgez@kau.edu.sa

For more morphology details, SEM-EDS images were provided in Figures S1, S2, S3, S4 and S5 for 0%, 10%, 20%, 30% and 40% Bi_2O_3 loadings into PMMA matrix respectively.

For more clarity and details, Figure S6 shows XRD spectra separately for all PMMA-Bi_2O_3 nanocomposites at 0%, 10%, 20%, 30% and 40% Bi_2O_3 loadings.
