# Supplementary figures and images for: Study of Prepared Lead-Free Polymer Nanocomposites for X- and Gamma-ray Shielding in Healthcare Applications
Source: Polymers (Basel). 2023 Apr 29;15(9):2142. doi: 10.3390/polym15092142 (PMC10181160; doi:10.3390/polym15092142)

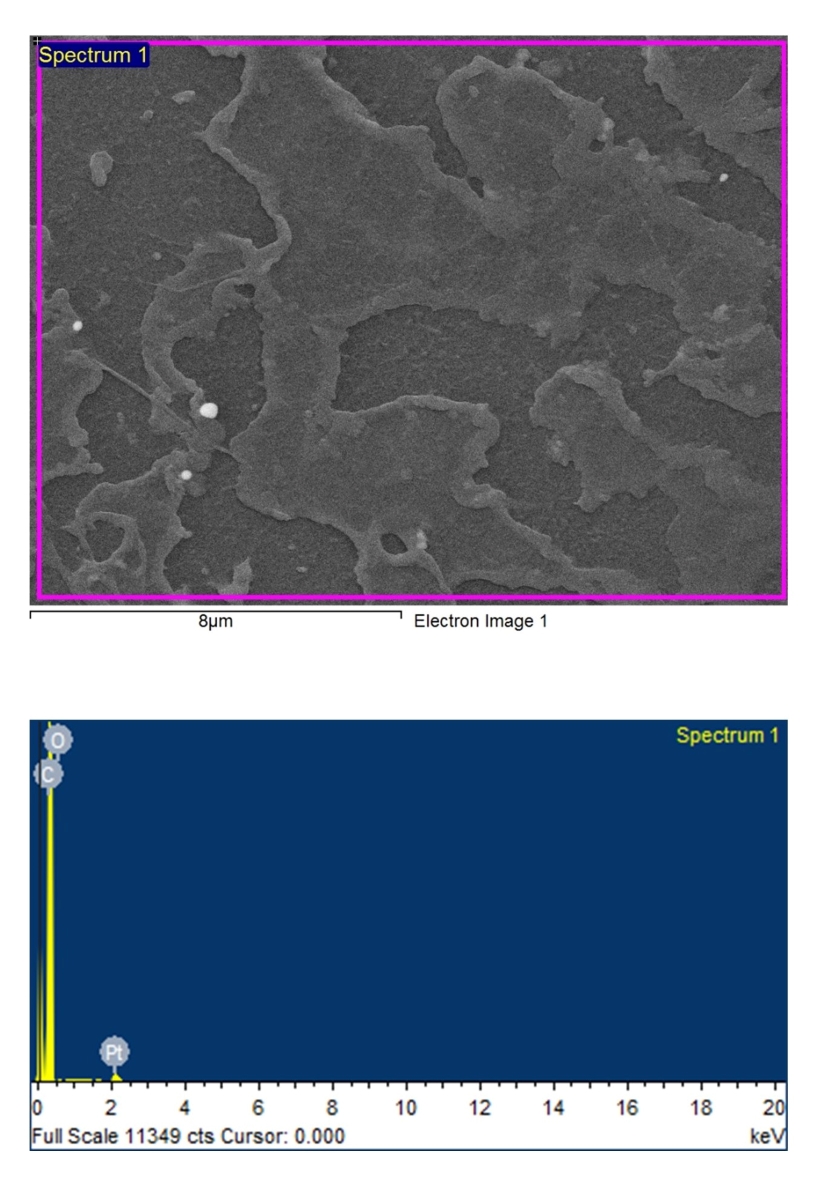

Supplement: Supplementary file 1 [file polymers-15-02142-s001.zip › Figure S1 SEM_EDS images of Pristine PMMA.jpg]

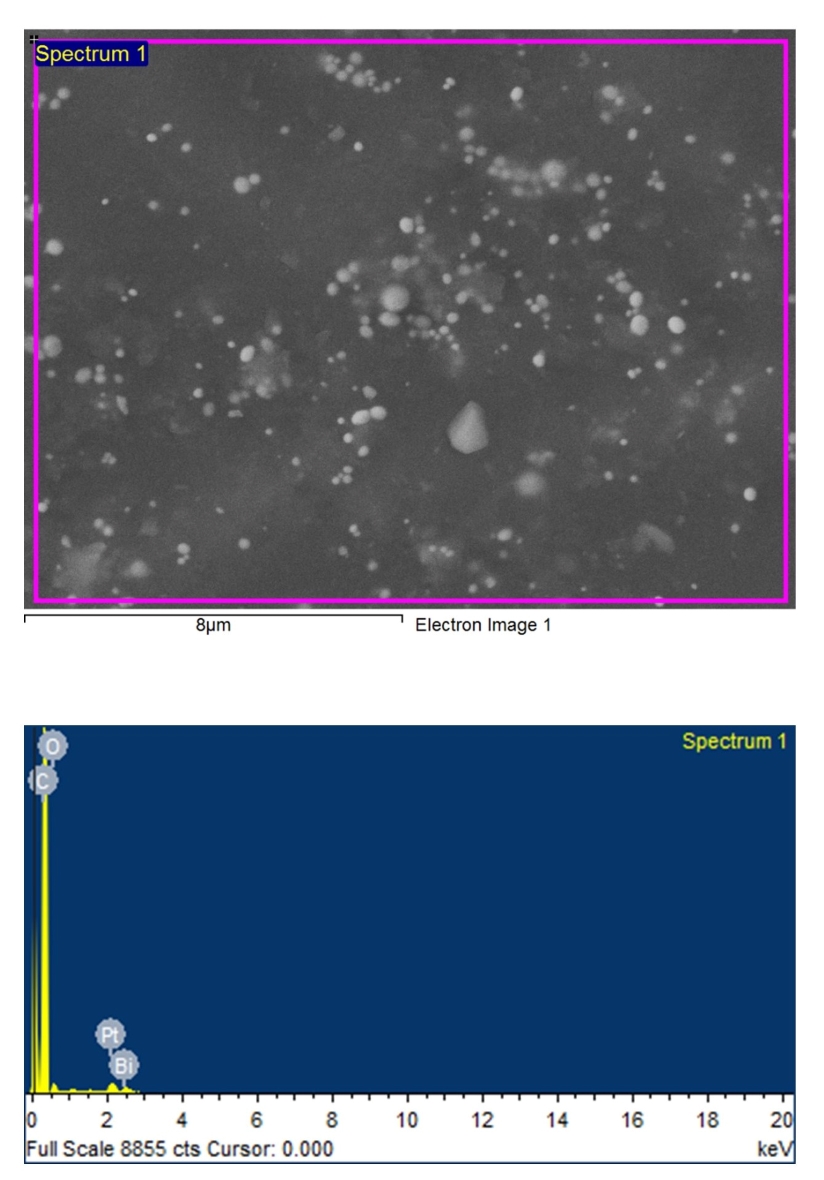

Supplement: Supplementary file 1 [file polymers-15-02142-s001.zip › Figure S2 SEM_EDS images of 10% loading of Bi2O3.jpg]

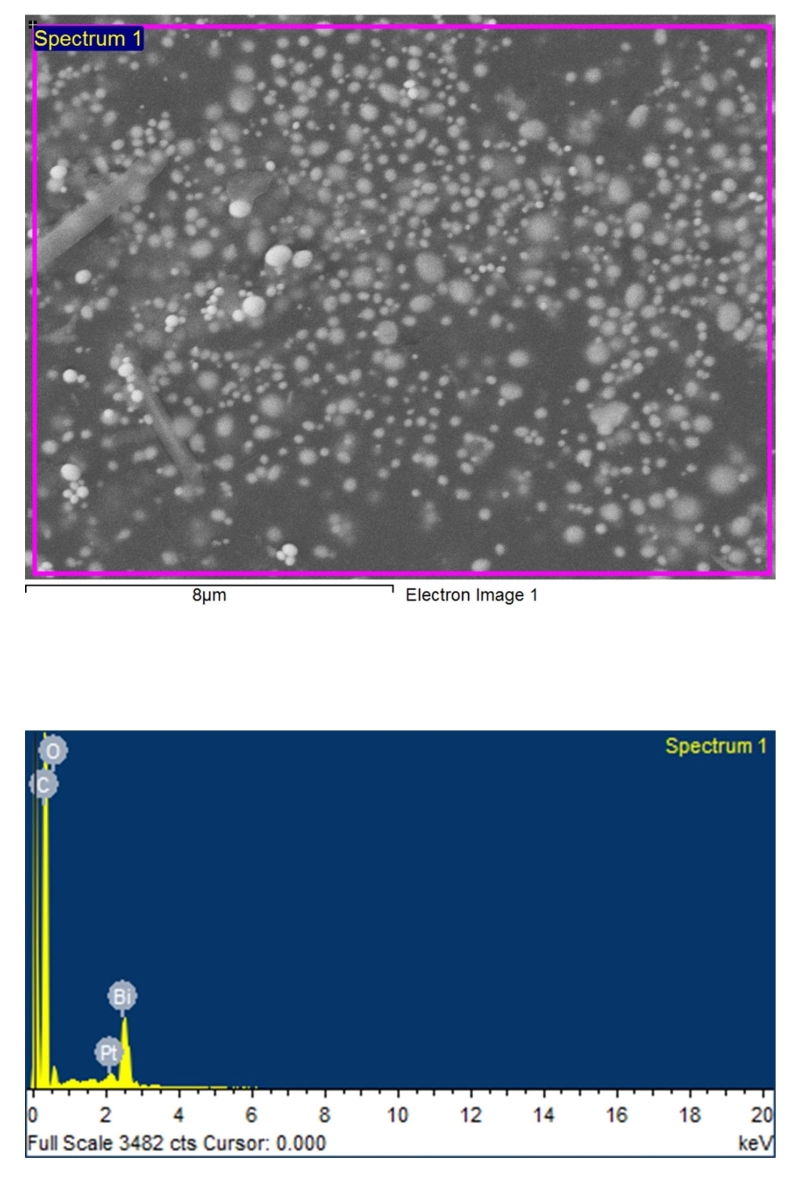

Supplement: Supplementary file 1 [file polymers-15-02142-s001.zip › Figure S3 SEM_EDS images of 20% loading of Bi2O3.jpg]

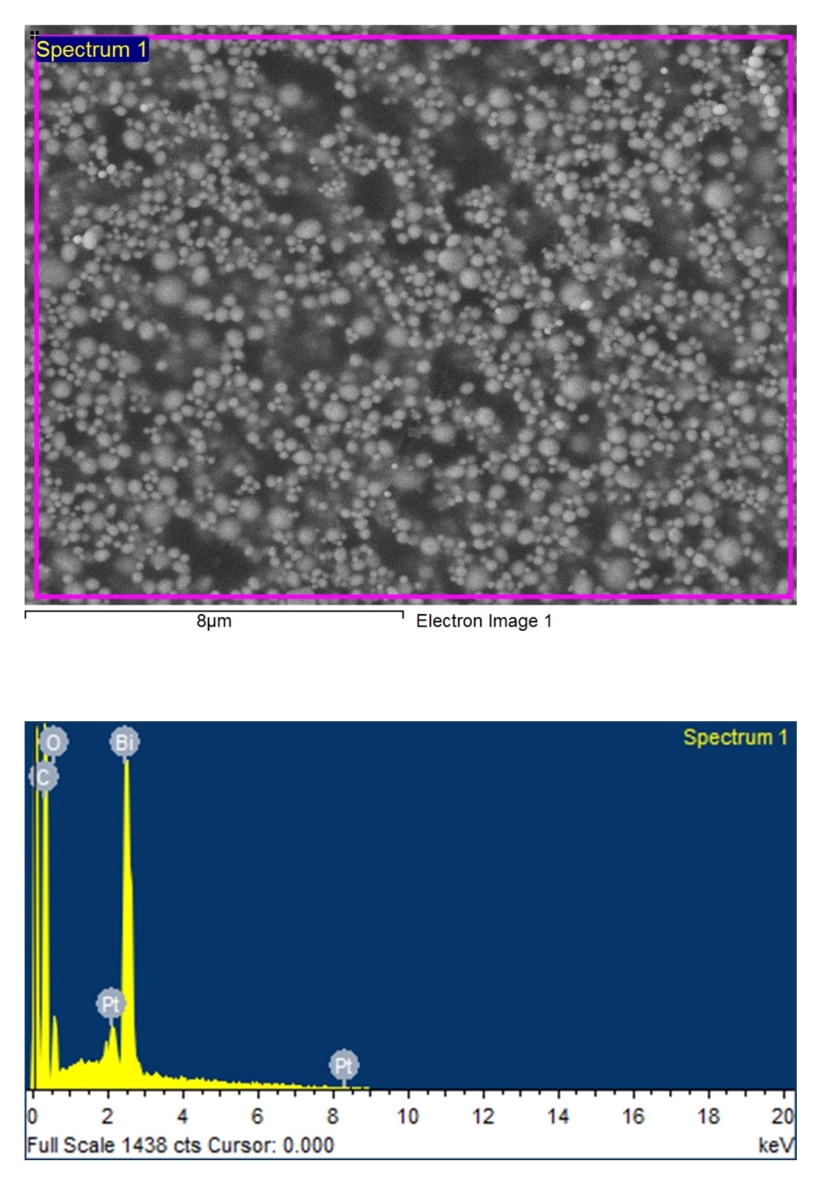

Supplement: Supplementary file 1 [file polymers-15-02142-s001.zip › Figure S4 SEM_EDS images of 30% loading of Bi2O3.jpg]

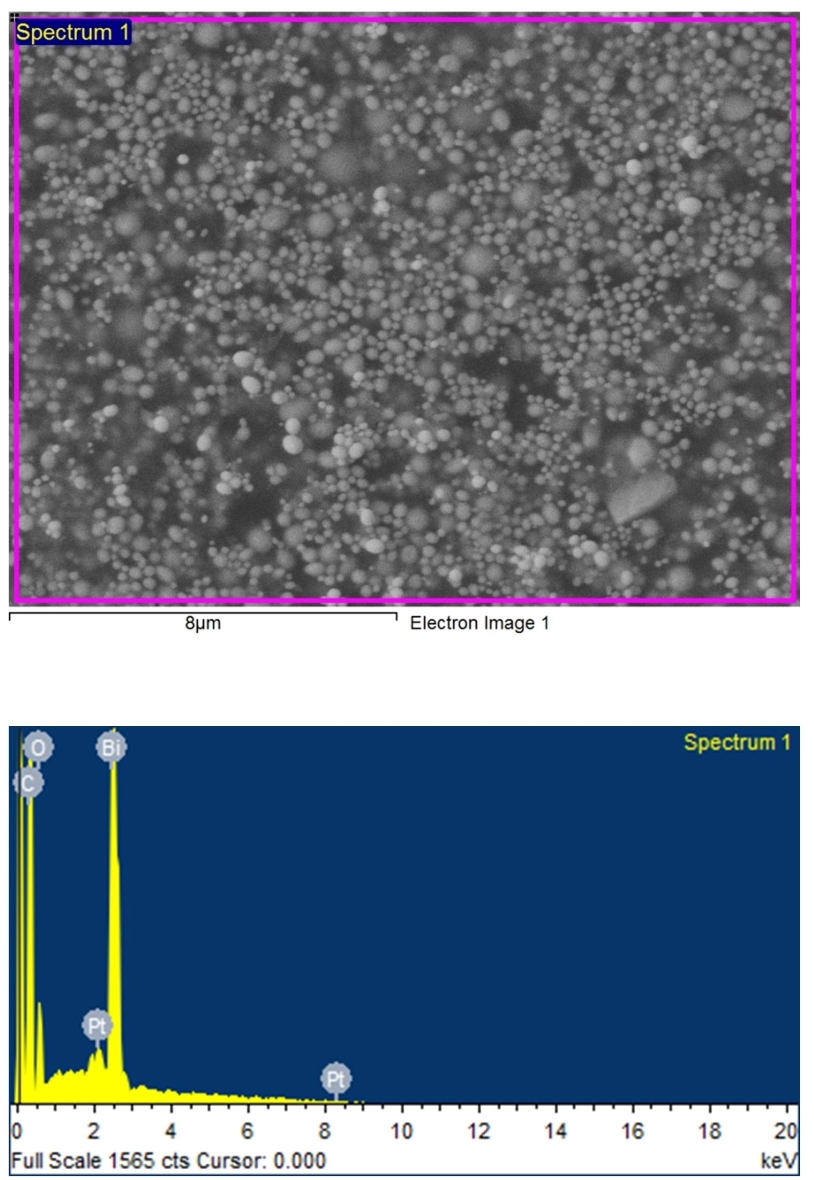

Supplement: Supplementary file 1 [file polymers-15-02142-s001.zip › Figure S5 SEM_EDS images of 40% loading of Bi2O3.jpg]

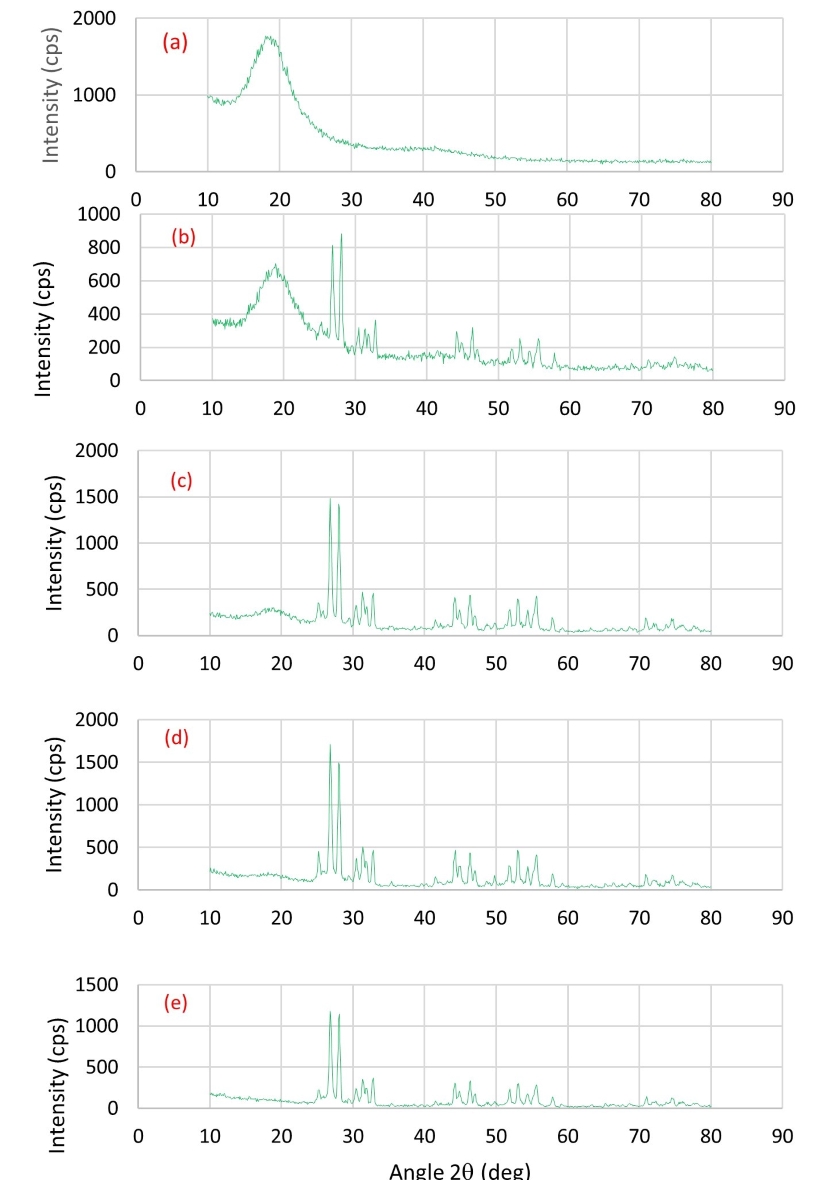

Supplement: Supplementary file 1 [file polymers-15-02142-s001.zip › Figure S6 XRD spectra separated for all PMMA-Bi2O3 nanocomposites.jpg]
